# Assessment of peripheral blood DNA methylation signatures as pharmacodynamic and predictive biomarkers during azacitidine therapy in juvenile myelomonocytic leukaemia: Results of the EWOG‐MESRAT study

**DOI:** 10.1111/bjh.70046

**Published:** 2025-07-31

**Authors:** Maximilian Schönung, Silvia Rathmann, Senthilkumar Ramamoorthy, Dirk Lebrecht, Thomas Klingebiel, Franco Locatelli, Karsten Nysom, Claudia Rossig, Jan Starý, Marco Zecca, Meera Patturajan, Miriam Erlacher, Brigitte Strahm, Charlotte M. Niemeyer, Daniel B. Lipka, Christian Flotho

**Affiliations:** ^1^ Section of Translational Cancer Epigenomics, Division of Translational Medical Oncology German Cancer Research Center (DKFZ) Heidelberg Germany; ^2^ National Center for Tumor Diseases (NCT), NCT Heidelberg, a Partnership between DKFZ and Heidelberg University Hospital Heidelberg Germany; ^3^ Department of Pediatric Hematology and Oncology, Faculty of Medicine Children's Hospital, Medical Center, University of Freiburg Freiburg Germany; ^4^ Faculty of Medicine, Institute of Medical Bioinformatics and Systems Medicine, Medical Center University of Freiburg Freiburg Germany; ^5^ Department of Pediatrics Frankfurt University Hospital Frankfurt Germany; ^6^ German Cancer Consortium (DKTK), Partner Site Frankfurt Frankfurt Germany; ^7^ Department of Pediatric Hematology and Oncology IRCCS Ospedale Pediatrico Bambino Gesu Rome Italy; ^8^ Department of Pediatrics Catholic University of the Sacred Heart Rome Italy; ^9^ Department of Paediatrics and Adolescent Medicine Copenhagen University Hospital – Rigshospitalet Copenhagen Denmark; ^10^ Department of Pediatric Hematology and Oncology University Children's Hospital Münster Münster Germany; ^11^ Department of Paediatric Hematology and Oncology Charles University and University Hospital Motol Prague Czech Republic; ^12^ Pediatric Hematology/Oncology Fondazione IRCCS Policlinico San Matteo Pavia Italy; ^13^ Bristol Myers Squibb Summit New Jersey USA; ^14^ Department of Pediatrics and Adolescent Medicine University Medical Center Ulm Ulm Germany; ^15^ German Cancer Consortium (DKTK), DKFZ, Core Center Heidelberg Heidelberg Germany; ^16^ German Cancer Consortium (DKTK), Partner Site Freiburg, a Partnership between DKFZ and University Medical Center Freiburg Freiburg Germany

**Keywords:** azacitidine, DNA methylation, epigenetics, JMML

## Abstract

EWOG‐MESRAT (European Working Group—*Me*thylation *S*ignatures and *R*esponse to *A*zacitidine *T*herapy; DRKS00007185) is an investigator‐initiated trial that studied EPIC array‐based DNA methylation patterns and next generation sequencing (NGS)‐based variant allele frequencies (VAFs) of driver mutations in peripheral blood (PB) and bone marrow (BM) of 11 patients with newly diagnosed juvenile myelomonocytic leukaemia (JMML) during therapy with azacitidine. We demonstrate that the pharmacodynamic activity of azacitidine can efficiently be monitored in PB and BM. DNA methylation subgroup classification was linked to clinical response after three cycles of azacitidine and found to be conserved between PB and BM in all patients. In contrast, neither changes in VAFs nor changes in DNA methylation patterns during the course of therapy correlated with therapy outcome among the 11 study patients. This work thus supports the value of DNA methylation subgroup classification from PB samples for response prediction of single‐agent azacitidine in patients with JMML.

## INTRODUCTION

Juvenile myelomonocytic leukaemia (JMML) is a myelodysplastic/myeloproliferative neoplasm with an incidence of approximately 1.2 per million children. RAS pathway mutations in the genes *PTPN11*, *NF1*, *KRAS*, *NRAS, CBL* or rarely *RRAS* and *RRAS2* are present in leukaemic cells of more than 95% of patients and define clinically and genetically distinct subtypes.^1^

JMML is a clinically highly heterogeneous entity and most patients require allogeneic haematopoietic stem cell transplantation (HSCT) for long‐term survival.^2^ The role of anti‐leukaemic therapy prior to HSCT remains unclear. Mercaptopurine, low‐dose cytarabine or fludarabine in combination with high‐dose cytarabine have been applied to control tumour burden. However, the response is usually transient, and there is limited evidence of pretransplant chemotherapy improving survival.^3^, ^4^

Azacitidine (5‐azacytidine, 5‐aza) is a DNA hypomethylating agent (HMA) that is incorporated into nucleic acids due to its structural similarity with cytidine.^5^ Several different antineoplastic mechanisms for azacitidine have been proposed, including direct stalling of DNA methyltransferases, interference with RNA metabolism, induction of apoptosis, promotion of anti‐tumour immunity and reactivation of endogenous retroviral elements.^6^, ^7^, ^8^, ^9^, ^10^, ^11^

Azacitidine (Vidaza®) was authorized by the European Medicines Agency in 2008 for adult patients with myelodysplastic syndromes (MDS), acute myeloid leukaemia (AML) or chronic myelomonocytic leukaemia (CMML).^12^ After several reports of favourable response and low toxicity of azacitidine given off‐label for JMML,^13^, ^14^ a prospective phase II multicentre trial sponsored by Celgene, a Bristol‐Myers Squibb Company, was conducted in Europe from 2015 to 2019 (study title AZA‐JMML‐001, EudraCT 2014‐002388‐13, NCT02447666). The primary objective was clinical complete or partial remission after three cycles of therapy; secondary objectives included safety, additional efficacy markers, pharmacodynamics and pharmacokinetics of azacitidine. The study enrolled 18 JMML patients; clinical partial remission was observed in 11 patients after three cycles of azacitidine and leukaemia‐free survival at the end of follow‐up (median 23.8 months) in 14 patients who underwent HSCT, alongside a good safety profile.^15^ Analysis of JMML DNA methylation subgroups showed that all patients assigned to low (LM) and intermediate (IM) methylation groups responded to azacitidine therapy.^15^, ^16^ The results of AZA‐JMML‐001 have subsequently enabled the approval of azacitidine monotherapy for newly diagnosed JMML by the Food and Drug Administration (FDA) in 2022.

EWOG‐MESRAT (European Working Group—*Me*thylation *S*ignatures and *R*esponse to *A*zacitidine *T*herapy) was an accompanying investigator‐initiated research study designed to investigate the dynamics of variant allele frequencies (VAFs) of RAS pathway driver and subclonal mutations, as well as DNA methylation patterns during azacitidine therapy administered in AZA‐JMML‐001. Specifically, we were interested to analyse whether the assessment of DNA methylation pattern and mutation frequency in peripheral blood (PB) cells correlated with the corresponding measurements in bone marrow (BM). This would be of immediate benefit to this vulnerable patient population by obviating the need for repetitive BM aspirates.

## METHODS

### Study design and patients

**FIGURE 1:**
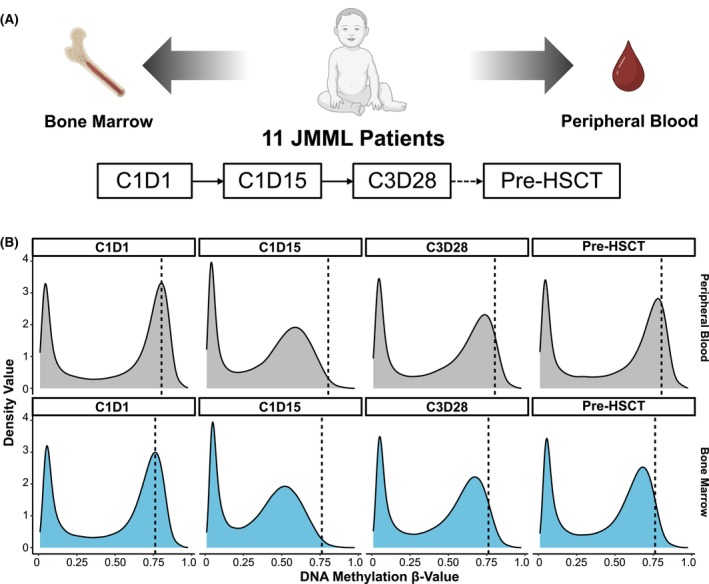
Study design and pharmacodynamics of azacitidine in juvenile myelomonocytic leukaemia (JMML). (A) We investigated DNA methylation patterns and VAFs of JMML driver mutations in peripheral blood (PB) and bone marrow (BM) of 11 JMML patients at different time points during therapy with azacitidine. (B) Density plots showing the distribution of mean DNA methylation *β*‐values across all patients in PB (grey; upper row) or BM (blue; lower row) at different study time points. Dashed lines indicate the peak position of methylated CpG sites (*β* > 0.5) at C1D1 for either PB or BM. C1D1, day 1 of cycle 1; C1D15, day 15 of cycle 1; C3D28, day 28 of cycle 3; pre‐HSCT, prior to allogeneic stem cell transplantation. VAF, variant allele frequency.

EWOG‐MESRAT is an investigator‐initiated study accompanying the AZA‐JMML‐001 trial.^15^ AZA‐JMML‐001 was industry‐sponsored and conducted between 2015 and 2019. The trial included children aged 1 month to less than 18 years with newly diagnosed JMML characterized by a somatic driver mutation in *PTPN11, KRAS, NRAS* and fetal haemoglobin >5× normal value for age, or a clinical diagnosis of neurofibromatosis type 1. Azacitidine was administered for a minimum of 3 and a maximum of six cycles on days 1–7 of a 28‐day cycle at a dose of 75 mg/m^2^ intravenously. For 11 of the 16 children who completed three cycles of azacitidine in the AZA‐JMML‐001 study, PB specimens were available. These 11 patients were included in the EWOG‐MESRAT study (Figure 1; Tables S1 and S2). BM and PB were sampled at cycle 1 day 1 (C1D1), cycle 1 day 15 (C1D15) and cycle 3 day 28 (C3D28) of azacitidine therapy and additionally before HSCT (pre‐HSCT). Clinical and molecular response was assessed according to the criteria defined in AZA‐JMML‐001.^15^

### Patient consent and ethics approval statement

EWOG‐MESRAT (German Clinical Trials Registry DRKS00007185) had been approved by the Ethics Committee of the University Medical Center of Freiburg (578/14 and 578/14_160208). Informed consent was obtained from legal representatives of the children, and study conduct conformed to the ethical principles for medical research stated in the Declaration of Helsinki.

### DNA methylation analysis

DNA methylation analysis was conducted at the Genomics and Proteomics Core Facility of the German Cancer Research Center (DKFZ) by subjecting 500 ng DNA to Infinium MethylationEPIC BeadChip array (EPIC arrays; Illumina) analysis. Data were processed using *RnBeads* ^17^, ^18^ with *R* version 3.5.1.^19^ Probes with missing values, an overlap with sex chromosomes or common single nucleotide polymorphisms (SNPs) were removed from the dataset, followed by p‐value filtering using the Greedycut algorithm with a *p*‐value threshold of 0.01. Data from the C1D1 time point (before azacitidine treatment) were normalized using beta‐mixture quantile (BMIQ) normalization and JMML DNA methylation subgroups determined according to the international consensus definition.^16^ To account for shifts in beta‐value distributions after azacitidine treatment, data involving longitudinal analysis over the different sampling time points were normalized using subset quantile within array normalization.

### VAF of JMML driver mutations and secondary mutations

DNA from BM or PB granulocytes was used for targeted next‐generation sequencing. The panel consisted of coding regions of *PTPN11* (NM_002834), *KRAS* (NM_ 004985), *NRAS* (NM_002524), *CBL* (NM_005188, exons 7–10), *NF1* (NM_001042492), *ASXL1* (NM_015338, exons 11–12), *JAK2* (NM_004972.3, exon 14), *JAK3* (NM_000215.3, exons 11–13,15,17,19), *RRAS* (NM_006270), *RRAS2* (NM_012250), *RAC2* (NM_ 002872), *RUNX1* (NM_001754), *SETBP1* (NM_015559, exon 4) and *SH2B3* (NM_005475, exons 2–7). Libraries were prepared using NEBNext Ultra II kits (New England Biolabs), and samples were sequenced on a MiSeq 2000 sequencer (Illumina) with 150 bp paired‐end reads. The reads were aligned to the human reference genome (hg19) using *bwa‐mem* (v0.7.17)^20^ and processed using the *GATK toolkit* (v3.8.1).^21^ Variants were identified using *samtools‐mpileup* (v1.9)^22^ and *VarScan* (v.2.4.3).^23^ Variants were annotated using *Annovar*,^24^ *Intervar* (v 2.0.2)^25^ and *SnpEff* (v4.3).^26^

## RESULTS

We studied PB from 11/18 (61%) patients of the AZA‐JMML‐001 study cohort at different time points of the treatment schedule (C1D1, C1D15, C3D28 and pre‐HSCT) by EPIC array‐based DNA methylation analysis and next generation sequencing (NGS)‐based VAF assessment of JMML driver mutations (Figure 1A). BM DNA methylation data and VAF data of the AZA‐JMML‐001 trial from matching time points had been kindly provided.^15^

The median age of patients enrolled was 2.2 years (min: 0.4; max: 3.4). 5/11 (45%) patients were female. The mutational subgroup was *PTPN11* in 8/11 (73%) patients, *NRAS* in 2/11 (18%) patients and *KRAS* in 1/11 (9%) patients. Secondary mutations affecting *CBL*, *JAK3* and *NF1* were detected in 3/11 (27%) patients. 7/11 (64%) patients were scored as clinical responders after three cycles of azacitidine by the criteria defined in AZA‐JMML‐001.

We observed a similar global reduction of DNA hypermethylation upon administration of azacitidine at C1D15 in PB as previously described in BM^15^ (Figure 1B; Figure S1A) with subsequent re‐methylation towards the end of treatment cycles (C3D28) and before HSCT, indicating that PB is similarly useful as BM to monitor the pharmacodynamic activity of azacitidine. Similar effects were observed for DNA methylation levels of JMML‐specific differentially methylated probes (DMPs); in both tissues, a reduction of DNA methylation levels at C1D15 and re‐establishment of DNA methylation levels at C3D28 and pre‐HSCT was observed (Figure S1B). Interestingly, both global and JMML‐specific DNA methylation patterns at pre‐HSCT were lower than at C1D1 (Figure 1B; dashed line), indicating that azacitidine treatment can have a long‐term demethylating effect in JMML.

**FIGURE 2:**
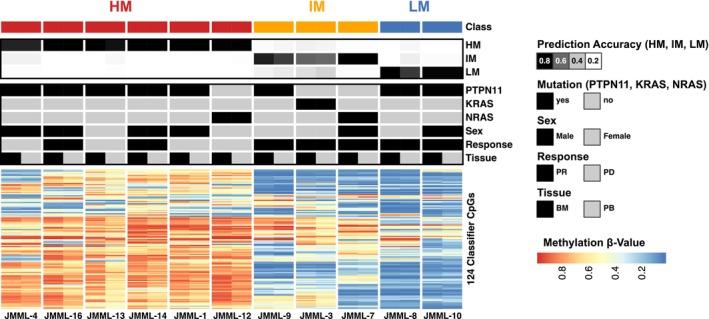
Juvenile myelomonocytic leukaemia (JMML) DNA methylation subgroups are conserved between bone marrow and peripheral blood. Heat map showing DNA methylation β‐values of the 124 classifier CpG sites assessed in BM and PB of the EWOG‐MESRAT patients. DNA methylation subgroups (Class), prediction accuracies for LM, IM and HM, mutations (*PTPN11*, *KRAS*, *NRAS*), tissue source (BM and PB), sex (male and female) and clinical response (PR and PD) at cycle 3 day 28 are annotated for each patient. Patients are separated by gaps, and the patient identifiers are annotated. BM, bone marrow; EWOG‐MESRAT, European Working Group—*Me*thylation *S*ignatures and *R*esponse to *A*zacitidine *T*herapy; HM, high methylation; IM, intermediate methylation; JMML, juvenile myelomonocytic leukaemia; LM, low methylation; PB, peripheral blood; PD, progressive disease; PR, partial response.

PB data of the C1D1 time point (before azacitidine therapy) were assigned to JMML DNA methylation subgroups using a previously published classifier^16^ and compared to DNA methylation subgroup assignments from BM (Table S1). Overall, 6/11 patients were thereby classified as high (HM), 3/11 as intermediate (IM) and 2/11 as low methylation (LM) JMML (Figure 2).

No conflicting classification between BM or PB samples of the same patient was observed. This was underlined by a high correlation of DNA methylation values for the 124 classifier CpGs (Figure S2), indicating that JMML DNA methylation subgroups can be reliably determined from PB samples prior to azacitidine therapy. As indicated by the results of study Aza‐JMML‐001, all patients from the LM (*n* = 2/2) and IM (*n* = 3/3) subgroups were responders whereas only 2/6 HM patients responded.

**FIGURE 3:**
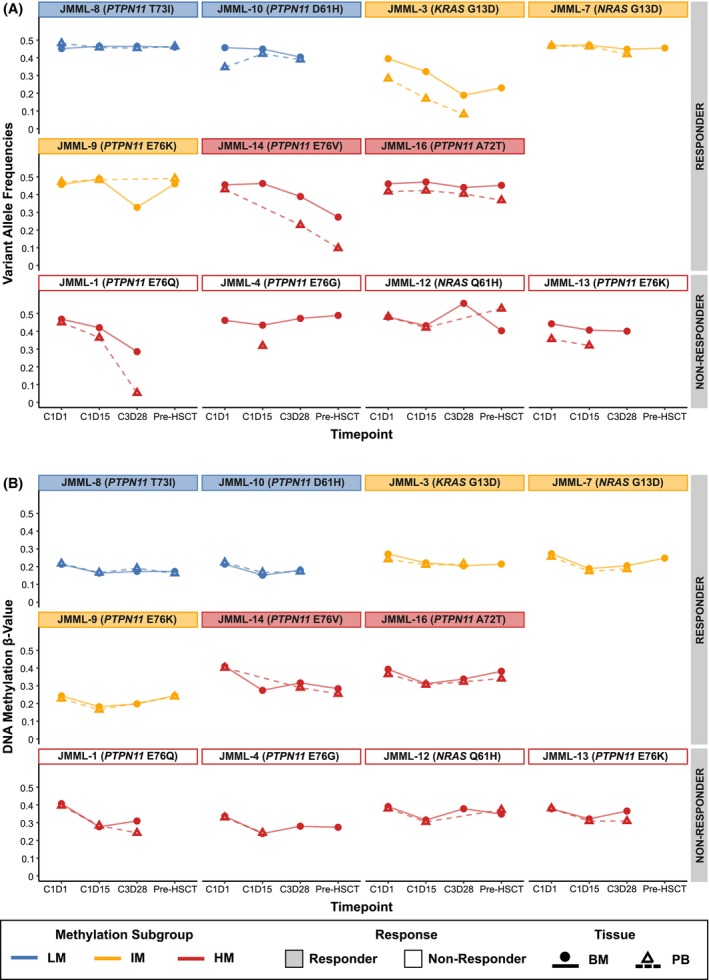
Molecular response to therapy with azacitidine in JMML. (A) Change in variate allelic frequency (VAF) of JMML driver mutations and (B) mean DNA methylation β‐values of JMML‐specific DMPs (*n* = 4920).^27^ Points with continuous lines display data from BM and triangles with dashed lines from PB. JMML DNA methylation subgroups are represented by box and line colours. Filled boxes denote patients with a clinical response. BM, bone marrow; HM, high methylation; JMML, juvenile myelomonocytic leukaemia; IM, intermediate methylation; LM, low methylation; PB, peripheral blood.

The correlation of JMML DNA methylation subgroups in PB with clinical response prompted us to investigate whether tissue changes in mutation burden or DNA methylation patterns during azacitidine therapy could be used as molecular biomarkers for response. We first analysed changes of VAFs across the study time points (Figure 3A; Table S3). The change in VAFs of JMML driver mutations between C1D1 and either C1D15 or C3D28 was greater than 10% in the BM of three of 11 patients and in the PB of three of 11 patients. None of the patients achieved a complete molecular response. Clinical partial response (PR) was observed in two of three patients with >10% VAF change. However, only three of seven patients with PR showed a reduction in VAFs of more than 10%. Collectively, these findings indicate that, in this study, cohort molecular monitoring of JMML driver mutations was not a useful predictor of response during azacitidine therapy in either PB or BM.

Others have linked the number of somatic variants in JMML cells (secondary mutations) to the risk of treatment failure.^28^ We therefore analysed in our study cohort whether patients with secondary genetic events might experience an inferior response to azacitidine. Using DNA panel sequencing, secondary mutations were detected in three of 11 patients, with no difference between BM and PB (Table S4). A PR to azacitidine therapy after three cycles was observed in two of three patients with and five of eight patients without secondary mutations.

Changes of more than 10% in mean DNA methylation of JMML‐DMPs between C1D1 and either C1D15 or C3D28 were observed in two of 11 patients; only one of these showed a PR after three cycles of azacitidine (Figure 3B; Tables S1 and S5). The VAF change in the other responders was in the range of −1% to +7% for BM and −4% to +5% for PB. We conclude that the extent of demethylation of JMML‐specific DMPs during the course of therapy is not predictive of response to azacitidine.

In summary, our study provides additional insight into the molecular monitoring of azacitidine therapy in JMML patients. We showed that the hypomethylating effect of azacitidine can be monitored in both PB and BM of the patients. Changes in VAFs or DNA methylation at JMML‐specific DMPs during three cycles of azacitidine therapy in these 11 patients were not consistently associated with response. Importantly, we showed that DNA methylation subgroup assignment, which determines the likelihood of response to azacitidine, is consistent between both PB and BM.

## DISCUSSION

HMAs have emerged as potent therapies for adult MDS and AML and became the standard of care for elderly AML patients not eligible for intensive chemotherapy.^29^ Additionally, response to azacitidine and safe application has been described for a subset of paediatric MDS patients and JMML.^13^, ^14^, ^30^, ^31^, ^32^, ^33^ Nevertheless, a systematic assessment of the effectiveness and safety in a clinical trial setting has been missing. This changed with the completion of the AZA‐JMML‐001 trial that described an acceptable safety profile for pre‐HSCT monotherapy with azacitidine in 18 JMML patients.^15^ Furthermore, a superior response for JMML patients of the LM and IM subgroup was noticed, in line with the more favourable risk profile in these subgroups observed in earlier retrospective analyses.^15^, ^16^, ^27^, ^34^ In the AZA‐JMML‐001 trial, DNA methylation subgroups and pharmacodynamics were determined from BM, requiring repetitive aspirates. Since this is not the standard of practice for JMML outside a clinical trial setting, we designed EWOG‐MESRAT to analyse PB in conjunction with BM collected at the same time point in the AZA‐JMML‐001 cohort.^15^ The correlation of DNA methylation in BM and PB at CpG sites included in the JMML DNA methylation classifier turned out to be extraordinarily high, meaning that the subgroups can be determined equally well from BM or PB data. Since JMML DNA methylation subgroups are significantly associated with overall survival and correlate with response to azacitidine, we thus show that a minimally invasive PB sample is sufficient for risk assessment in JMML. Our work thereby demonstrates how an investigator‐initiated trial can provide additional insights for patient benefit when conducted in parallel to industry sponsored studies.

Various reports for a spectrum of myeloid malignancies have established DNA methylation signatures at the time point of diagnosis that are predictive of response to HMAs.^35^, ^36^, ^37^ It is likely that these signatures reflect different disease subgroups that differ in therapeutic response to azacitidine, similar to what is observed here in JMML. Besides epigenetic patterns, mutational signatures were also described that correlated with HMA response in patients.^38^, ^39^ However, the relevance of these signatures when translated into different patient cohorts was questionable.^40^

We have observed in EWOG‐MESRAT that neither changes in JMML‐specific DNA methylation levels nor in VAFs of JMML driver mutations during the course of azacitidine therapy are associated with response. Similarly, the response of CMML patients to HMAs was shown to be independent of changes in the mutational allele burden whereas it rather affected gene expression and global DNA methylation.^41^ From the small cohort of 11 JMML patients presented here, we cannot exclude the possibility that a VAF reduction of specific JMML driver mutations might well be associated with response to azacitidine. We observed that this is at least not the case for the majority of patients and hotspot mutations. Likewise, distinct DNA methylation signatures after treatment with azacitidine might exist that could function as biomarkers for response. However, the definition of these would require additional validation cohorts and larger sample sizes.

In summary, the EWOG‐MESRAT study provides evidence that JMML DNA methylation subgroups determined from PB DNA samples serve as valuable diagnostic markers for the risk of treatment failure and thus obviate the need for repetitive BM punctures in these paediatric patients.

## AUTHOR CONTRIBUTIONS

M. S., S. Rath, S. Ram, D. L., D. B. L. and C. F. collected, analysed and interpreted data. S. Rath and C. F. designed and administered the study. T. K., F. L., K. N., C. R., J. S., M. Z., M. P., M. E., B. S., C. M. N. and C. F. collected and provided patient and study material. M. S., S. Rath, D. B. L. and C. F. wrote the initial draft of the manuscript. All authors critically revised and approved the manuscript.

## FUNDING INFORMATION

This study was supported by the German Research Foundation (CRC 992‐C05 and SPP1463 FL345/4‐2). M.S. was supported by the Joachim Herz Foundation.

## CONFLICT OF INTEREST STATEMENT

M. P. is an employee of Bristol Myers Squibb. D. B. L. receives honoraria from Infectopharm GmbH. The other authors declare no conflicts of interest.

## PERMISSION TO REPRODUCE MATERIAL FROM OTHER SOURCES

DNA methylation data from BM were adopted from the AZA‐JMML‐001 trial with permission and reanalysed for this study.^15^

## CLINICAL TRIAL REGISTRATION

EWOG‐MESRAT was registered at the German Clinical Trials Registry DRKS00007185.

## Supporting information


Data S1.

Data S2.

## Data Availability

DNA methylation EPIC array and targeted next‐generation sequencing data that support the findings of this study are available from the corresponding authors upon request.
