# Supplementary material for: Assessment of peripheral blood DNA methylation signatures as pharmacodynamic and predictive biomarkers during azacitidine therapy in juvenile myelomonocytic leukaemia: Results of the EWOG‐MESRAT study
Source: Br J Haematol. 2025 Jul 31;207(4):1271–8. doi: 10.1111/bjh.70046 (PMC12512093; doi:10.1111/bjh.70046)
Supplement: Supplementary file 1 — Data S1. [file BJH-207-1271-s002.docx]

**Supplementary Figure 1 | DNA methylation dynamics during the course of azacitidine therapy**. A) Violin plots displaying the distribution of DNA methylation β-values for 100,000 randomly selected CpG sites at different timepoints of azacitidine therapy stratified by patient and tissue. B) Boxplots display the mean DNA methylation β value of 4920 JMML-specific DMPs for each patient stratified by patient, timepoint and tissue. Interquartile range (IQR) and median are shown as a box. Whiskers are defined as 1.5 times IQR. BM, bone marrow; PB, peripheral blood.

**Supplementary Figure 2 | Correlation of JMML DNA methylation subgroup classifier CpGs in BM and PB**. Pearson correlation between PB and BM DNA methylation data for the 124 classifier CpG sites was calculated and plotted as bar for each patient. Bars are colored by DNA methylation class. LM, low methylation; IM, intermediate methylation; HM, high methylation.
